# Supplementary material for: Dual-Channel Stopped-Flow Apparatus for Simultaneous Fluorescence, Anisotropy, and FRET Kinetic Data Acquisition for Binary and Ternary Biological Complexes
Source: Biosensors (Basel). 2020 Nov 19;10(11):180. doi: 10.3390/bios10110180 (PMC7699296; doi:10.3390/bios10110180)
Supplement: Supplementary file 1 [file biosensors-10-00180-s001.pdf]

biosensors

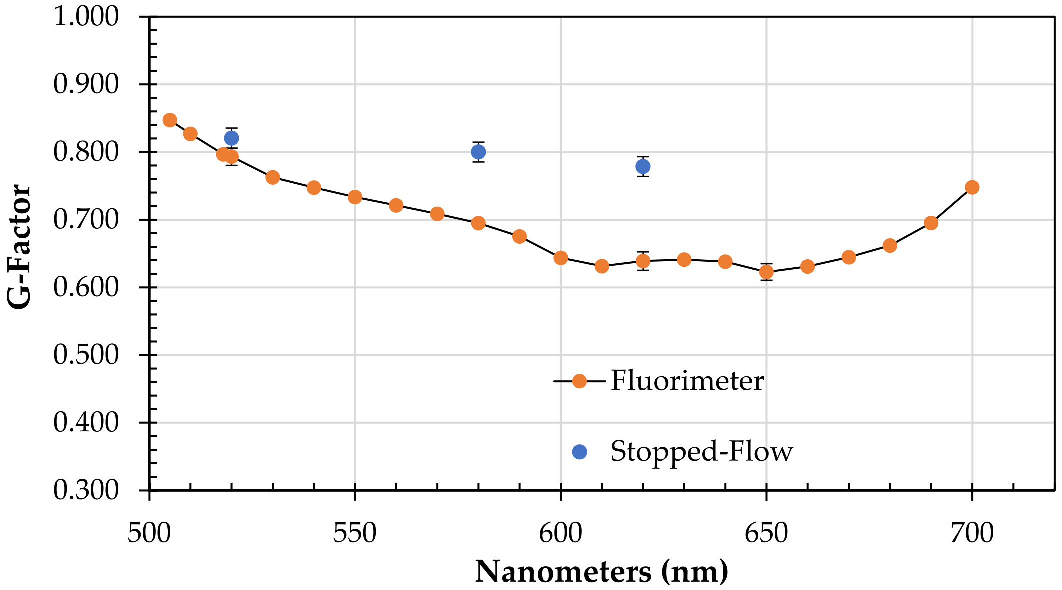

Figure S1. The grating-factor of the fluorimeter and the stopped-flow. The SF G-factor (blue dots) was obtained by triplication through the 520-nm, 580-nm, and 620-nm interference filters and a polarizer at the vertical and horizontal position to acquire the I_{HV}/I_{HH} ratio with scans of 3-5 secs. The excitation Ar+ laser and sample concentrations were those described in the experimental section of each of the association reactions. The fluorimeter's G-factor (orange dots) was obtained from 500-700 nm by measuring the I_{HV}/I_{HH} ratio acquired by polarizers with 3-5 s scans carried out by triplicated with a step size of 5-10 nm and the emission monochromator bandwidth set at 1 turn. The fluorimeter *G*-factor calculations required an $AdMLP_{14ds}$*Fl solution at a concentration of 0.5 µM, to yield the values in the 500-590 nm range. The fluorimeter *G*-factor in the 580-700 nm range was acquired by a solution of Xr*$AdMLP_{14ds}$ at a concentration of 4.7 µM at excitations of 510 nm and 560 nm.

© 2020 by the authors. Submitted for possible open access publication under the terms and conditions of the Creative Commons Attribution (CC BY) license (http://creativecommons.org/licenses/by/4.0/).
